# Non-Thermal Ultrasonic Extraction of Polyphenolic Compounds from Red Wine Lees

**DOI:** 10.3390/foods9040472

**Published:** 2020-04-09

**Authors:** Filip Dujmić, Karin Kovačević Ganić, Duska Ćurić, Sven Karlović, Tomislav Bosiljkov, Damir Ježek, Rajko Vidrih, Janez Hribar, Emil Zlatić, Tihomir Prusina, Sucheta Khubber, Francisco J. Barba, Mladen Brnčić

**Affiliations:** 1 Laboratory for Thermodynamics, Faculty of Food Technology and Biotechnology, University of Zagreb, Pierottijeva 6, 10000 Zagreb, Croatia; filip.dujmic@pbf.unizg.hr; 2 Laboratory for Technology and Analysis of Wine, Faculty of Food Technology and Biotechnology, University of Zagreb, Pierottijeva 6, 10000 Zagreb, Croatia; kkova@pbf.unizg.hr; 3 Laboratory for Cereal Chemistry and Technology, Faculty of Food Technology and Biotechnology, University of Zagreb, Pierottijeva 6, 10000 Zagreb, Croatia; duska.curic@pbf.unizg.hr; 4 Laboratory for Unit Operations, Faculty of Food Technology and Biotechnology, University of Zagreb, Pierottijeva 6, 10000 Zagreb, Croatia; sven.karlovic@pbf.unizg.hr (S.K.); tomislav.bosiljkov@pbf.unizg.hr (T.B.); damir.jezek@pbf.unizg.hr (D.J.); 5 Department of Food Science and Technology, Biotechnical Faculty, University of Ljubljana, Jamnikarjeva 101, 1000 Ljubljana, Slovenia; rajko.vidrih@bf.uni-lj.si (R.V.); janez.hribar@bf.uni-lj.si (J.H.); emil.zlatic@bf.uni-lj.si (E.Z.); 6 Čitluk Winery dd, Kralja Tomislava 28, 88260 Čitluk, Bosnia and Herzegovina; tiho@vinarija-citluk.ba; 7 Food Engineering and Nutrition, Center of Innovative and Applied Bioprocessing, Mohali 140306, Punjab, India; suchetakkr@gmail.com; 8 Nutrition and Food Science Area, Preventive Medicine and Public Health, Food Science, Toxicology and Forensic Medicine Department, Faculty of Pharmacy, Universitat de València, Avda. Vicent Andrés Estellés, 46100 Burjassot, València, Spain

**Keywords:** non-conventional ultrasound, wine lees, extraction parameters, HPLC, antioxidants

## Abstract

This study presents the results of conventional aqueous (CE) and non-conventional ultrasound-assisted (UAE) extractions of polyphenolic compounds from lees extracts of red wine varieties (Merlot and Vranac). The effect of ultrasound extraction time (t, s), and amplitude (A,%) from a 400 W ultrasound processor with different ultrasonic probes diameters (Ds, mm) on the amount and profile of polyphenolic compounds in the obtained extracts was investigated and compared to CE. The optimal conditions resulting in maximum extraction of phenolic compounds were: Probe diameter of 22 mm, amplitude 90% and extraction time for Vranac wine lees 1500 s and for Merlot wine lees extraction time of 1361 s. UAE proved to be significantly more effective in enhancing the extraction capacity of trans-resveratrol glucoside (30.57% to 300%), trans-resveratrol (36.36% to 45.75%), quercetin (39.94% to 43.83%), kaempferol (65.13% to 72.73%), petunidin-3-glucoside (41.53% to 64.95%), malvidin-3-glucoside (47.63% to 89.17%), malvidin-3-(6-*O*-acetyl) glucoside (23.84% to 49.74%), and malvidin-3-(6-*O*-p-coumaroyl) glucoside (26.77% to 34.93%) as compared to CE. Ultrasound reduced the extraction time (2.5-fold) and showed an increase of antioxidant potential by 76.39% (DPPH) and 125.83% (FRAP) compared to CE.

## 1. Introduction

Grapes are one of the fruit crops produced in largest quantities throughout the world. About 80% of all grapes are used in wine production [1]. The annual production of 60 million tons of grapes is mostly for wine production, which consequently generates huge amounts of by-products such as stalks, pomace, seeds, and lees. These by-products are potentially good sources of valuable bioactive compounds. Wine lees are the sediment remaining in vessels containing wine after fermentation and during wine maturation [2]. According to the EEC regulation 337/79 wine lees are defined as residues formed at the bottom of vessels containing wine, after fermentation, during storage, as well as the residue obtained following filtration or centrifugation [3]. It is a known fact that the skin and seeds of black grape berries (*V. vinifera* L.), as well as red wines naturally contain more than 200 different polyphenolic compounds [4,5,6,7,8,9]. Previous studies performed on extraction of polyphenolic compounds from red grapes were mostly focused on their extraction from seeds or the epidermis of grape berries. Highly profitable commercial extracts of polyphenolic compounds originating from grapes can be found in the market [9,10,11,12].

Scientific studies have confirmed that polyphenolic compounds have a positive impact on human health, primarily due to their antioxidant effects that protect the body from harmful radicals [13,14,15,16,17]. Higher consumption of phenolic antioxidants (accomplished through moderate consumption of wine, especially red wine) correlates to a decreased incidence of certain coronary heart diseases [18,19,20,21,22,23]. The most relevant phenolic and polyphenolic compounds in red grapes are tannins, anthocyanins, flavanols, flavonols, and stilbenes of which most notable are the resveratrols, phenolic acids and their derivatives [24,25,26,27,28,29,30].

An important compound in red wine containing particular antioxidant properties is resveratrol (3,5,4‘-trihydroxy-stilbene) [31,32,33]. A very important class of polyphenolic compounds in wines are the flavonoids, which constitute >85% of the total phenol content in red wines. Among these are anthocyanidins, which are viewed as having a strong antioxidant effect and procyanidins, which are even stronger in terms of antioxidant efficiency in wine [34,35,36,37].

Polyphenol rich residues of red wines may be used to enrich several food products, thus attracting the interest of food producers [38,39,40,41,42,43]. Grape polyphenols can be extracted successfully using conventional extraction (maceration) [44] or modern innovative extraction methods [45] such as high-intensity ultrasound-assisted extraction [46,47], microwave-assisted extraction [48], high pressures (i.e., supercritical extraction) [49], or pulsed electric field extraction [50]. Besides traditional technologies like extrusion, freezing, distillation and drying, ultrasound has found applications in the food processing and bioactives extraction in the food industry and biotechnology [51,52,53,54]. The application of high intensity ultrasound has proven to be extremely effective as a pretreatment for drying [55,56], emulsification [57], and for other uses in the food industry and biotechnology [58,59].

An innovative high-intensity ultrasound process has proven to be highly effective for extracting polyphenolic compounds [40,60,61,62,63,64,65,66,67,68], and with reduced amount of solvents (enabling the use of green solvents and solvents permitted for human consumption). Moreover, these technologies have been reported to be relatively friendly to the environment [69,70]. The efficiency of ultrasound-assisted extraction of polyphenolic compounds depends on various factors such as frequency, rated output power, amplitude, probe geometry, treatment time, temperature, dry matter content, sample particle size and type of solvent used [71,72,73].

Recently, natural deep eutectic solvents (NADESs) such as choline chloride:malic acid (ChMa), choline chloride:oxalicacid (ChOa), and choline chloride:citric acid (ChCit) have been recognized as a novel class of sustainable solvents to replace common organic solvents. Combination of ultrasound-assisted extraction and natural deep eutectic solvents (NADESs) of wine lees anthocyanins to result higher efficiency of extraction have also been previously explored [74].

Despite numerous potential applications, large quantities of lees generated as a by-product during the production of red wines are discarded [45,73,75,76,77]. Therefore, the present study aims to analyze the polyphenols extracted from wine lees found in grape varieties Merlot and Vranac the common regional variety grown in Croatia, as well as to explore the possibility of using UAE for enhancing the extraction efficiency of high value polyphenolic compounds. 

## 2. Materials and Methods 

### 2.1. Chemicals and Reagents 

Ethanol from Pharmachem (Ljubljana, Slovenia), formic acid from Sigma-Aldrich Chemie GmbH (Schnelldorf, Germany) and deionized water, purified using a Milli-Q water system (Millipore, Burlington, USA) were used to prepare the extracts. Standards including malvidin 3-*O*-glucoside, petunidin 3-*O*-glucoside, procyanidin B1 and procyanidin B2; were purchased from Polyphenols (Sandnes, Norway); while kaempferol, (+) catechin, (-) epicatechin, myricetin, trans-resveratrol quercetin and isorhamnetin were obtained from Sigma-Aldrich Chemie GmbH (Schnelldorf, Germany) for identification and quantification utilizing HPLC–mass spectrometry (MS) and HPLC– diode array detection (DAD).

All chromatographic solvents (HPLC grade) and the remaining reagents (analytical or high-performance liquid chromatography (HPLC) grade) were obtained from Sigma-Aldrich Chemie GmbH (Schnelldorf, Germany). The solutions were prepared using Milli-Q water (Millipore, Burlington, USA). All sample preparations, extractions and chemical analyses were carried out at Biotechnical Faculty University of Ljubljana, Department of Food Science, Slovenia. 

### 2.2. Samples

The present study utilized wine lees from two varieties of red grapes i.e., Merlot grape varieties grown in Istria (Croatia) and Vranac grape variety grown in Mostar, (Bosnia and Herzegovina). The wine lees were sampled and immediately packed in impermeable polyethylene bags and frozen. Lees were then lyophilized, and subsequently packed in vacuum bags and stored at −80 °C before extraction.

### 2.3. Preparation of Wine Lees for Extraction

Thawed samples of lyophilized lees were crushed in the mortar and sieved through 500 microns sieve. Samples were prepared in a 200 mL of 50% aqueous ethanol (*v***/***v*) mixture containing 1.5% formic acid (*v***/***v*). The ratio of the dry matter to solvent was 1:60 (*w/v*) as previously described [73].

### 2.4. Extraction of Bioactive Compounds from Wine Lees

#### 2.4.1. Conventional Extraction 

Extraction of previously prepared wine lees samples as described was carried out using an aqueous bath (temp. 25 °C) with the external stirrer (40 rpm), for 1 h, as described [45,78,79].

#### 2.4.2. High-Intensity Ultrasound-Assisted Extraction (UAE)

Ultrasound-assisted extraction was carried out using ultrasonic equipment UP 400s, procured from Laboratory of Thermodynamics, Faculty of Food Technology and Biotechnology, University of Zagreb, Croatia. UAE of bioactive substances in the wine lees (Cw) was performed using ultrasonic processor with nominal power of 400 W at a constant frequency of 24 kHz. Five different amplitudes (A), 30%, 38.79%, 60%, 82.21%, and 90%, and treatment times (t) of 120 s, 322.10 s, 810 s, 1297.90 s, and 1500 s, with ultrasonic probes of diameter (Ds) 22 mm and 40 mm, at a full cycle were considered as optimization conditions in accordance with the central composite rotatable design (CCRD) for the experiments. Following extraction under these conditions, 50 mL of each extract was centrifuged at 4000 rpm/15 min. to separate the lees particles as residues. The extracts were then flushed with inert nitrogen gas and stored in dark at −80 °C till further analyses. The results obtained represent the mean value of three replicates.

### 2.5. Bioactive Potential of Extracted Compounds

#### 2.5.1. Ferric Reducing Antioxidant Power (FRAP) Assay

A standard solution of Trolox 100 mg/L was prepared in methanol and working solutions of 25−500 μM were used. Briefly, 0.1 mL of extracts (diluted 1:10) was mixed with 3 mL of a FRAP reagent (25 mL of acetate buffer 300 mM at pH = 3.6 (corrected with formic acid) + 2.5 mL of Fe (II)-TPTZ 10 mM in HCl 40 mM + 2.5 mL of FeCl_3_ × 6H_2_O, 20 mM). The FRAP reagent was used as a blank, with final absorbance read at 593 nm after 10 min at room temperature. The ferric reducing antioxidant power of the samples (AOP _FRAP_) was determined in triplicate and expressed as mg of Trolox equivalents per gram of the dried wine lees sample (mg TEAC/g d.m.) [80,81,82,83]. A calibration curve was freshly prepared before each assay using 5-point calibration plot.

#### 2.5.2. 2-Diphenyl-1-picrylhydrazyl (DPPH) Assay

The method utilizes scavenging potential of 2,2-diphenyl-1-picrylhydrazyl (DPPH) radical with an absorbance maximum at 515 nm. The radical is reduced in reaction with an antioxidant or another radical [84]. Determination of the antioxidant activity was carried out according to [85]. Briefly, the extracts were diluted (1:5, *v*/*v*) and subsequently 100 μL of the diluted extracts was added to 2.9 mL of a methanol solution of the radical DPPH (with a concentration of 6 × 10^-5^ M) and an absorbance of 515 nm was measured after 25 min at room temperature. A calibration line (5 points plot) was freshly prepared before each assay from methanol solutions of Trolox ranging from 0.19 to 0.93 mM [85]. The results (AOP_DPPH_) were expressed as mg of Trolox equivalents per gram of samples of dried wine lees (mg TEAC/g d.m.).

### 2.6. Determination of Total Phenolic Content

The total phenolic compounds content (TPC) of the samples were determined using the Folin–Ciocalteu assay and the results were expressed as mg/g dry matter (d.m.) of gallic acid equivalents (GAE) [86,87,88], with some modifications. Briefly, Milli-Q water was added to aliquots of the extracts (diluted 1:5, *v*/*v*) in order to obtain a final volume of 1.400 mL and then mixed with 300 μL of freshly prepared Folin–Ciocalteu reagent diluted with water (1:2, *v*/*v*). The mixtures were vortexed and allowed to react for 5 min. Then, 300 μL of 20% sodium carbonate in water (*w*/*v*) was added and the tubes were vortexed. After 60 min of incubation, the absorbance was measured in a 1-cm cuvette at 765 nm using a UV–vis spectrophotometer (CECIL CE 2021, 2000 Series, Cecil Instruments Limited, Cambridge, UK) at a room temperature [89]. The results were expressed as mg of GAE/gram of dried wine lees. All the samples were measured in triplicate.

### 2.7. HPLC-DAD-ESI-MS/MS Analyses for Phenolic Characterization

Polyphenol analysis was performed according to method described by Bosiljkov et al. [74] and modified with the following elution gradient: solvent B: 0–20 min, 14–23%; 20–40 min, 23–35%; 40–50 min, 40–60 min, 60%; 60–65 min 95%. Method in brief description analysis was carried out using LC-ESI-MS/MS an Agilent 1260 series LC and Agilent LC-QQQ-MS G6460A mass spectrometer (Agilent Technologies, Palo Alto, CA, USA) equipped with an electrospray ionization (ESI) interface. The LC system includes a G1322A on-line degasser, a G1312B Bin Pump, a G1367E autosampler, a G1330B thermostatic column control, and a G4218B DAD, all of which were controlled by the Agilent MassHunter B 6.0 software. The HPLC separation was performed on a Poroshell 120 EC-C18 column (120 × 2.1 mm i.d. 2.7 μm particle size, Agilent Technologies, Palo Alto, CA, USA) at 30 °C. The mobile phase consisted of 1% formic acid in water (solvent A) and methanol (solvent B) by applying the following gradient: 0−20 min: 2−23% B, 20−40 min: 23−35% B, 40−46 min: 35−38% B, 46−60 min: 60% B, 60−65 min: 95% B. The flow rate was 0.2 mL min^-1^. The injection volume was 1.0 μL with the UV detector set to an absorbance wavelength of 280 nm for phenolic acid, 520 nm for anthocyanins and 360 nm for flavonol glycosides. The mass spectrometer was equipped with electrospray ion source (ESI), parameters were as follows: nebulizer 35 psi; dry gas (N2) flow, 6 L min^−1^; and dry gas temp. 300 °C; capillary voltage, 4 kV where the ion trap mass spectrometer was operated in negative/positive ion mode with a scanning range from m/z 100 to m/z 1000. Individual phenolic compounds were identified by comparing their retention times MS/MS and UV/Vis spectra with those of authentic standards [89,90,91]. Quantification of phenolic compounds were calculated from the peak areas of the samples and corresponding standards. For the compounds lacking standards, the quantification was achieved using similar compounds. Trans-resveratrol glucoside was quantified in equivalents of trans-resveratrol, malvidin-3-(6-*O-p*-coumaroyl) glucoside and malvidin-3-(6-*O*-acetyl) glucoside were quantified in equivalents of malvidin 3-*O* glucoside. 

### 2.8. Experimental Design and Statistical Analysis 

A central composite rotatable design for the UAE experiments based on two numeric factors: A (%) and t (s), set to five levels i.e., ±1.414 (axial points), ±1 (factorial points), center point (with five replicates for both probes and both wine lees varieties); and two categorical factors (±1): Ds (mm) and wine less cultivar (Cw) was prepared using the statistical software Design-Expert 9.0.6. (Stat-Ease, MN, USA). 

The reduced quadratic model equation Equation (1) was used to express the investigated responses (Yn) as a function of the coded independent variables (A, t, Ds, and Cw), where a_0_ and a_1_-a_23_ represent the intercept and regression coefficients for linear, quadratic, and interaction effects, respectively.

Y_n_ = a_0_ + a_1_ × A + a_2_ × t + a_3_ × D_s_ + a_4_ × C_w_ + a_5_ × A × t + a_6_ × A × D_s_ + a_7_ × A × C_w_ + a_8_ × t × D_s_ + a_9_ × t × C_w_ + a_10_ × D_s_ × C_w_ + a_11_ × A_2_ + a_12_ × t_2_ + a_13_ × A × t × D_s_ + a_14_ × A × t × C_w_ + a_15_×A+D_s_×C_w_ + a_16_×t × D_s_ × C_w_ + a_17_ × A_2_ × D_s_ + a_18_ × A_2_ × C_w_ + a_19_ × t_2_ × D_s_ + a_20_ × t_2_ × C_w_ + a_21_ × A × t × D_s_ × C_w_ + a_22_ × A_2_ × D_s_ × C_w_ + a_23_ × t_2_ × D_s_ × C_w_ (1)

The obtained results were statistically analyzed using ANOVA and backward elimination regression at the significance level of *p* < 0.05. The adequacy of the models was evaluated based on the coefficient of determination (R^2^) and the model p-value. The intercept (a_0_) in the acquired models represents the mean value of the investigated dependent variable (Yn) under the conditions of the performed experiments, whereas a_1-23_ refers to the significant regression coefficients which enable determination of the most significant effects of the investigated variables (*p*-value and numeric value) and their position and negative impact (sign + or -) on the investigated dependent variable. Response surface plots were generated using the design expert and were based on a function of two factors while keeping the others constant. Numerical and graphical optimization was carried out according to conditions for each response. Results of numerical optimization are described with desirability. Desirabilities range from zero to one for any given response. A value of one represents the case where all goals of optimization are met perfectly. A zero indicates that one or more responses fall outside desirable limits [92].

## 3. Results

The average measured values of all investigated variables in wine lees extracts obtained using conventional extraction and UAE are given in Table 1. The regression equations of polyphenolic compounds identified and quantified in extracts using HPLC-MS/MS with the significant coefficients for the studied effects of UAE conditions are given below in text. 

### 3.1. Antioxidant Potential and Total Phenolic Content Extracts

The antioxidant potential (AOP) obtained after using 2-diphenyl-1-picrylhydrazyl (DPPH) assay of wine lees extracts from conventional extraction was 44.21 and 49.72 mg trolox equivalent antioxidant capacity (TEAC)/g d.m. for the varieties Merlot and Vranac, respectively (Table 1). Moreover, from Table 1 it can be depicted a positive effect of UAE on the AOP. From the obtained regression equation Equation (2), it was observed that in the extracts obtained after UAE, the amplitude of ultrasonic processors and treatment time with linear and quadratic effect had a significant positive effect (*p* < 0.0001) on the AOP_DPPH_ of extracts whereas increasing the diameter of the ultrasound probe had a negative and linear effect on AOP_DPPH_ (*p* < 0.0001). As it is evident in Equation (2) the AOP_DPPH_ of wine lees extract is a varietal characteristic (*p* < 0.0001).On the other hand, the AOP obtained with ferric reducing antioxidant power (FRAP) assay of wine lees extracts after conventional extraction 42.52 and 50.39 mg TEAC/g d.m. for Merlot and Vranac varieties (Table 1). It should be noted that UAE also had a positive effect on the AOP.

AOP_DPPH_ = 64.73 + 3.25 × A + 7.50 × t − 4.880 × Ds + 1.34 × Cw − 2.74 × t^2^ (*p* < 0.0001, R^2^ = 0.9437) (2)

From the obtained regression equation Equation (3), it is evident that for AOP_FRAP_ there are significant linear and quadratic effects of the investigated variables as is for AOP_DPPH_ (*p* < 0.0001), the AOP_FRAP_ is subject to a significantly positive effect of the interrelation of A × t (*p* < 0.0414) and A^2^ × Ds (*p* < 0.0034), including the quadratic effect of A (*p* < 0.4394), whereas a negative effect is provided by the interrelation A × Ds (*p* < 0.0124), t × Ds (*p* < 0.0294), Ds × Cw (*p* < 0.0016) and quadratic effect of t (*p* < 0.0001).

AOP_FRAP_ = 74.22 + 4.58 × A + 10.27 × t − 7.50 × Ds + 2.11 × Cw + 1.66 × A × t − 1.46 × A × Ds − 1.26 × t × Ds − 1.48 × Ds × Cw + 0.47 × A^2^ −3.77 × t^2^ + 1.85 × A^2^ × D (*p* < 0.0001, R^2^ = 0.9476) (3)

The extracts of wine lees obtained after conventional extraction were found to contain a significant amount of total phenols (TPC_Merlot_ = 32.95 and TPC_Vranac_ = 41.22 mg GAE/g d.m., respectively) (Table 1). From Table 1 it was also observed that UAE had a positive effect on TFC. Equation (4) shows that there are significant effects of the investigated variables from the UAE of wine lees on the quantity of total phenols (TPC) found in the Merlot and Vranac extracts. On the TPC had a positive linear effect of t (*p* < 0.2530), Ds (*p* < 0.0019), Cw (*p* < 0.5570), interrelation A × t (*p* < 0.0029), A × Ds (*p* < 0.5090), A × Cw (*p* < 0.8688), t × Ds (*p* < 0.293), t × Cw (*p* < 0.0003), A^2^ × Ds × Cw (*p* < 0.0393). The TPC decreased with an increase in A (*p* < 0.3539), with the interrelation of Ds × Cw (*p* < 0.0346), A × Ds × Cw (*p* < 0.1570), A^2^ × Ds (*p* < 0.744), A^2^ × Cw (*p* < 0.0012) and A^2^ (*p* < 0.0151).

TPC = 47.25 + −0.69 × A + 0.86 × t + 2.53 × Ds + 0.45 × Cw + 3.33 × A × t + 0.49 × A × Ds + 0.123 × A × Cw × D + 1.68 × t × Ds + 2.98 × t × Cw − 1.68 × Ds × Cw − 2.00 × A^2^ − 1.06 × A × Ds × Cw − 0.26 × A^2^ × Ds −2.77 × A^2^ × Cw + 1.68 × A^2^ × Ds × Cw (*p* < 0.0001, R^2^ = 0.6963) (4)

**Figure 1:**
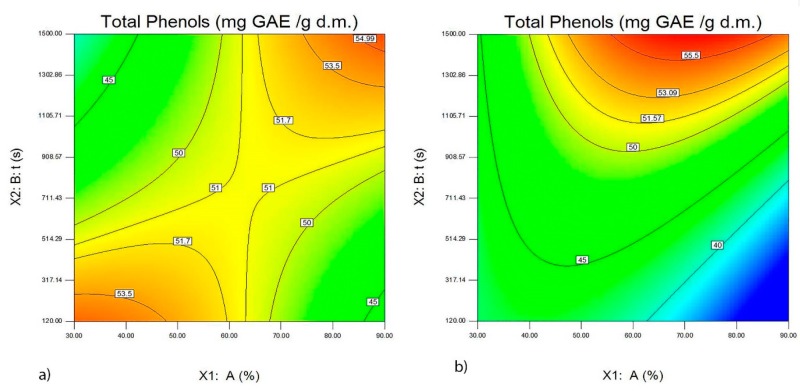
The effect of the probe with a diameter of 40 mm, the amplitude A (%), and ultrasound extraction time t (s), on the quantity of total phenols (mg GAE/g d.m.) in the wine lees extracts from the Merlot variety (**a**) and wine lees from the Vranac variety (**b**).

The presented Equation (4) and Figure 1a,b show that the greatest amount of total phenols is obtained in the extracts after using a probe of 40 mm at a middle amplitude and at different times of ultrasonic treatment depending on the origin of the wine lees. Wine lees from the Vranac variety contained a larger amount of total phenols.

### 3.2. Individual Phenolics of CW and UAE Extracts

#### 3.2.1. Trans-Resveratrol Glucoside

Equations (5) and (6) show that the most important effect on the quantity of t-Res-3-*O*-glc was provided by Cw (*p* < 0.0001), meaning that the quantity of these compounds is a characteristic of wine lees variety. Regarding the amount of t-Res-3-*O*-glc in the lees obtained using UAE, a positive linear effect was observed using A (*p* < 0.0001), t (*p* < 0.0001), Ds (*p* < 0.0001) and quadratic impact of t (*p* < 0.3025). As well significant positive impact was observed from the interrelations A × Ds × Cw (*p* < 0.0336), t × Ds × Cw (*p* < 0.0001), A^2^ × Cw (*p* < 0.0249), A^2^ × Ds × Cw (*p* < 0.0004) and t^2^ × Ds × Cw (*p* < 0.0410). A negative linear effect on the quantity of t-Res-3-*O*-glc was provided by Cw (*p* < 0.0001), as well as a quadratic effect from A (*p* < 0.0350) and interrelations A × Ds (*p* < 0.0160), A × Cw (*p* < 0.0001), t × Ds (*p* < 0.0001), t × Cw (*p* < 0.0001), Ds × Cw (*p* < 0.6957), A^2^ × Ds (*p* < 0.0002), t^2^ × Ds (*p* < 0.0293) t^2^ × Cw (*p* < 0.3766).

t−Res-3-*O*-glc = 0.34 + 0.02 × A + 0.01 × t + 0.00 × Ds − 0.33 × Cw − 0.00 × A × Ds − 0.01 × A × CW − 0.01 × t × Ds − 0.01 × t × Cw − 0.00 × Ds × Cw −0.00 × A^2^ + 0.00 × t^2^ + 0.00 × A × Ds × Cw + 0.01 × t × Ds × Cw − 0.01 × A^2^ × Ds + 0.00 × A^2^ × Cw − 0.00 × t^2^ × Ds − 0.00 × t^2^ × Cw + 0.01 × A^2^ × Ds × Cw + 0.00 × t^2^ × Ds × Cw (*p* < 0.0001, R^2^ = 0.9997) (5)

t-Res = 0.01 + 0.00 × A + 0.00 × t − 0.01 × Ds + 0.03 × Cw − 0.00 × A × Ds + 0.00 × A × Cw − 0.00 × t × Ds + 0.00 × t × Cw − 0.00 × Ds × Cw + 0.00 × A^2^ + 0.00 × t^2^ − 0.00 × A × Ds × Cw + 0.00 × A^2^ × Ds + 0.00 × A^2^ × Cw + 0.00 × t^2^ × Ds + 0.00 × t^2^ × Cw + 0.00 × A^2^ × Ds ×Cw (*p* < 0.0001, R^2^ = 0.9844) (6)

The quantity of *trans*-resveratrol (t-Res) was subject to a significant positive linear effect from A (*p* < 0.0359), t (*p* < 0.0003) and Cw (*p* < 0.0001), quadratic A (*p* < 0.0180) and t (*p* < 0.0134), including the interrelations A × Cw (*p* < 0.3058), t × Cw (*p* < 0.0042), A^2^ × Ds (*p* < 0.0474), A^2^ × Cw (*p* < 0.0004), t^2^ × Ds (*p* < 0.0408), t^2^ × Cw (*p* < 0.0349), A^2^×Ds×Cw (*p* < 0.0300). The quantity of extracted t-Res was subject to a negative effect from Ds (*p* < 0.0001) and the interrelation A × Ds (*p* < 0.4459), t × Ds (*p* < 0.0252), Ds × Cw (*p* < 0.0006), A × Ds × Cw (*p* < 0.3178).

**Figure 2:**
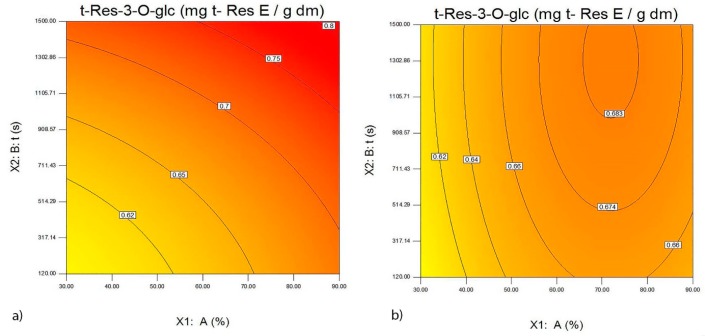
The effect of the probe with a diameter of 22 mm (**a**), probe with a diameter of 40 mm (**b**), amplitude A (%) and the ultrasound extraction time t (s) on the quantity of *trans*-resveratrol glucoside expressed as the *trans*-resveratrol equivalent (mg/g d.m.) in the extracts of wine lees from the Merlot variety.

**Figure 3:**
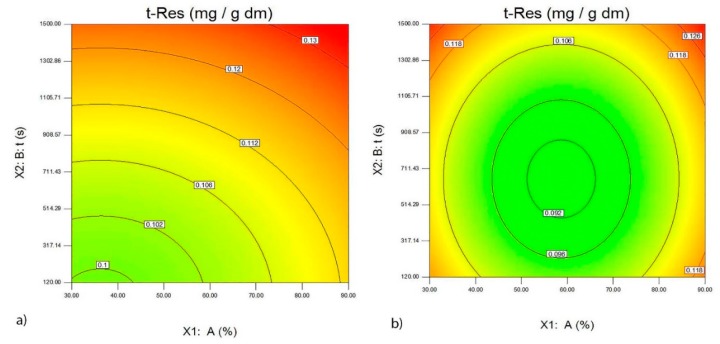
The effect of the probe with a diameter of 22 mm (**a**), probe with a diameter of 40 mm (**b**), amplitude A (%) and the time of ultrasound extraction t (s) on the quantity of *trans*-resveratrol (mg/g d.m.) in the extracts of the wine lees of the Vranac variety.

In the analyzed extracts of wine lees from the Merlot variety, the maximum quantity of t-Res-3-*O*-glc was obtained at high amplitudes and long extraction time when using the probe with a diameter of 22 mm (Figure 2a,b). The greatest quantity of t-Res was obtained in the extracts of wine lees from the Vranac variety, at high amplitudes and long extraction time when using the probe with a diameter of 22 mm (Figure 3a,b).

#### 3.2.2. Quercetin and Kaempferol

**Figure 4:**
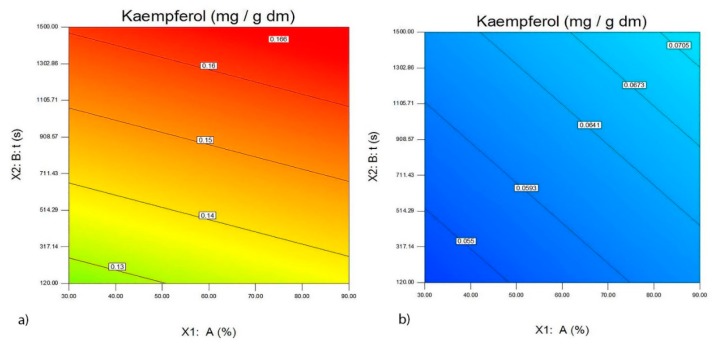
The effect of the probe with a diameter of 22 mm, amplitude A (%) and the time of ultrasound extraction t (s) on the quantity of Kaempferol (mg/g d.m.) in the extracts of the wine lees of the Merlot variety (**a**) and the wine lees of the Vranac variety (**b**).

Equation (7) shows the significant effects of the investigated variables of UAE for wine lees of the Merlot and Vranac variety on the quantity of extracted quercetin in the obtained extracts. The same equation indicates that a positive linear effect on the quantity of quercetin in lees is obtained after using UAE parameters A (*p* < 0.0079), t (*p* < 0.0001) and Cw (*p* < 0.6653), including the quadratic effect of t (*p* < 0.6362) and interrelations A × Cw (*p* < 0.0616), t × Cw (*p* < 0.0457), A × Ds × Cw (*p* < 0.0210), t × Ds × Cw (*p* < 0.1689), A^2^ × Cw (*p* < 0.0001), t^2^ × Ds (*p* < 0.1143), t^2^ × Cw (*p* < 0.6340), A^2^ × Ds × Cw (*p* < 0.0001), t^2^ × Ds × Cw (*p* < 0.0265). A negative linear effect on the quantity of quercetine in the lees was provided by Ds (*p* < 0.0001) and a quadratic effect by A (*p* < 0.4833) and the interrelations A × Ds (*p* < 0.0001), t × Ds (*p* < 0.0001), Ds × Cw (*p* < 0.0002), A^2^ × Ds (*p* < 0.0051). The analyzed extracts showed that the largest quantity of quercetin for both varieties was obtained using high amplitudes and long extraction times when using a probe with a diameter of 22 mm. Equation (8) shows the significant effects of the investigated variables of UAE for wine lees of the Merlot and Vranac on the quantity of extracted kaempferol in the obtained extracts. The same equation indicates that a positive linear effect on the quantity of kaempferol in lees is obtained using UAE with A (*p* < 0.0079), t (*p* < 0.0001), and interrelations between A × Cw (*p* < 0.0616). A negative linear effect on the quantity of kaempferol in the lees was provided by Ds (*p* < 0.0069) and Cw (*p* < 0.0001), including the interaction between t × Ds (*p* < 0.0069), t × Cw (*p* < 0.2954) and Ds × Cw (*p* < 0.6695). The analyzed extracts showed that the largest quantity of kaempferol for both varieties was obtained using high amplitudes and long extraction times when using a probe with a diameter of 22 mm (Figure 4a,b). Equation (8) and Figure 4, as well as the results, showed that the quantity of kaempferol is a varietal characteristic and that the lees of the Merlot contain two and half times more kaempferol.

Quercetin = 1.19+ 0.02 × A + 0.05 × t − 0.06 × Ds + 0.00 × Cw − 0.03 × A × Ds + 0.012 × A × Cw −0.03 × t × Ds + 0.01 × t × Cw − 0.03 × Ds × Cw − 0.01 × A^2^ + 0.00 × t^2^ + 0.01 × A × Ds × Cw + 0.01 × t × Ds × Cw − 0.02 × A^2^ × Ds + 0.03 × A^2^ × Cw + 0.01 × t^2^ × Ds+ 0.00 × t^2^ × Cw + 0.04 × A^2^ × Ds × Cw + 0.02 × t^2^ × Ds × Cw (*p* < 0.0001, R^2^ = 0.9241) (7)

Kaempferol = 0.10 + 0.00 × A + 0.00 × t − 0.00 × Ds − 0.04 × Cw − 0.00 × t × Ds − 0.00 × t × Cw −0.00 × Ds × Cw + 0.00 × t × Ds × Cw (*p* < 0.0001, R^2^ = 0.9797) (8)

#### 3.2.3. Petunidin-3-*O*-Glucoside

Equation (9) shows the significant effects of the investigated variables of UAE for wine lees of the Merlot and Vranac variety on the quantity of extracted Pt-3-glc in the obtained extracts. The conclusion from Equation (9) is that the quantity of Pt-3-glc in the extracts obtained using UAE is subject to a positive linear effect from A (*p* < 0.0098), t (*p* < 0.0011), whereas a linear negative effect on the quantity of extracted Pt-3-glc is provided by Ds (*p* < 0.0002) and Cw (*p* < 0.0001). The analyzed extracts showed that the largest quantity of Pt-3-glc for both varieties was obtained using high amplitudes and long extraction times when using a probe with a diameter of 22 mm.

Petunidin-3-*O*-glucoside = 2.08 + 0.07 × A + 0.10 × t − 0.09 × Ds − 0.82 × Cw (*p* < 0.0001, R^2^ = 0.9676) (9)

#### 3.2.4. Malvidin-3-Glucoside

Equation (10) shows a significant effect of the investigated variables of UAE on wine lees of the Merlot and Vranac regarding the amount of extracted malvidin-3-glucoside (Mv-3-glucoside) in the obtained extracts. The same equation indicates a positive linear effect on the quantity of malvidin-3-glucoside in lees after using UAE parameters A (*p* < 0.0009), t (*p* < 0.0001) and Cw (*p* < 0.0245), including the quadratic effect of A (*p* < 0.0312), t (*p* < 0.1852), and interactions A × Cw (*p* < 0.1879), t×Cw (*p* < 0.3030), A × Ds × Cw (*p* < 0.7606), A^2^ × Ds (*p* < 0.0061), A^2^ × Cw (*p* < 0.0058), t^2^ × Ds (*p* < 0.0086), t^2^ × Cw (*p* < 0.0143) and A^2^ × Ds × Cw (*p* < 0.0071). A negative linear effect on the quantity of Mv-3-glucoside in the wine lees from the Merlot and Vranac varieties was provided by Ds (*p* < 0.0001) and the interactions of A × Ds (*p* < 0.0705), t × Ds (*p* < 0.0245) and Ds × Cw (*p* < 0.0001).

**Figure 5:**
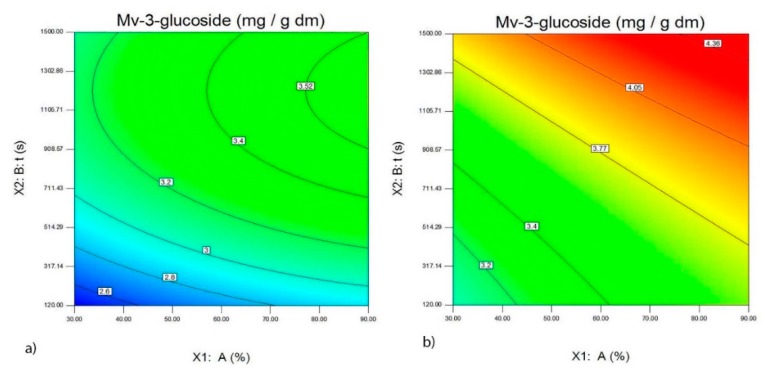
The effect of the probe with a diameter of 22 mm, amplitude A (%) and the time of ultrasound extraction t (s) on the quantity of Mv-3-glucoside (mg/g d.m.) in the extracts of the wine lees of the Merlot variety (**a**) and the wine lees of the Vranac variety (**b**).

The greatest quantities of Mv-3-glucoside in the analyzed extracts for both varieties were obtained at high amplitudes and long extraction times when using a probe with a diameter of 22 m (Figure 5a,b). Moreover, it is also evident that the extracts of wine lees from the Vranac variety contained greater amounts of Mv-3-glucoside.

Malvidin-3-glucoside = 3.18 + 0.12 × A + 0.18 × t − 0.35 × Ds + 0.05 × Cw − 0.06 × A×Ds + 0.04 × A × Cw − 0.08 × t × Ds + 0.03 × t × Cw − 0.14 × Ds × Cw + 0.08 × A^2^ + 0.05 × t^2^ + 0.01 × A × Ds × Cw + 0.10 × A^2^ × Ds + 0.10 × A^2^ × Cw + 0.10 × t^2^ × Ds + 0.09 × t^2^ × Cw + 0.10 × A^2^ × Ds × Cw (*p* < 0.0001, R^2^ = 0.8379) (10)

Equation (11) shows the significant effects of the investigated variables of UAE for wine lees of the Merlot and Vranac variety on the quantity of extracted malvidin-3-(6-*O*-acetyl) glucoside (Mv-acetyl-3-glc) in the obtained extracts.

The same equation indicates that a positive linear effect on the quantity of malvidin-3-(6-*O*-acetyl) glucoside in lees is obtained after using UAE parameters t (*p* < 0.0617), as well as a quadratic effect of t (*p* < 0.0312) and a significant positive effect due to the interrelations A × Cw (*p* < 0.0799), t × Cw (*p* < 0.6348), A × Ds × Cw (*p* < 0.1639), t × Ds × Cw (*p* < 0.8928), A^2^ × Cw (*p* < 0.0001), t^2^ × Ds (*p* < 0.0204), t^2^ × Cw (*p* < 0.8610) and A^2^ × Ds × Cw (*p* < 0.0024). A negative linear effect on the quantity of extracted malvidin-3-(6-*O*-acetyl) glucoside from the wine lees of the Merlot and Vranac varieties was provided by A (*p* < 0.9113), Ds (*p* < 0.0015), Cw (*p* < 0.0001), A^2^ (*p* < 0.3868) and the interrelations A × Ds (*p* < 0.0158), t × Ds (*p* < 0.1784), Ds × Cw (*p* < 0.2999), A^2^ × Ds (*p* < 0.2802) and t^2^ × Ds × Cw (*p* < 0.2577). The analyzed extracts showed that the largest amount of malvidin-3-(6-*O*-acetyl) glucoside was obtained for the Vranac variety when high amplitudes, long extraction times and a probe with a diameter of 22 mm were used, whereas for the extracts from the Merlot variety similar or greater amounts of malvidin-3-(6-*O*-acetyl) glucoside were obtained when lower amplitudes and shorter treatment times were used.

Malvidin-3-(6-*O*-acetyl) glucoside = 1.41 − 0.00 × A + 0.05 × t − 0.12 × Ds − 0.19 × Cw − 0.07 × A×Ds + 0.05 × A × Cw − 0.04 × t × Ds + 0.01 × t × Cw − 0.04 × Ds × Cw − 0.03 × A^2^ + 0.04 × t^2^ + 0.04 × A × Ds × Cw + 0.00 × t × Ds × Cw − 0.03 × A^2^ × Ds + 0.13 × A^2^ × Cw + 0.07 × t^2^ × Ds+ 0.01 × t^2^ × Cw + 0.10 × A^2^ × Ds × Cw − 0.03 × t^2^ × Ds × Cw (*p* < 0.0001, R^2^ = 0.7747) (11)

Equation (12) shows the significant effects of the investigated variables of UAE for wine lees of the Merlot and Vranac variety on the quantity of extracted malvidin-3-(6-*O*-*p*-coumaroyl) glucoside (Mv-3-*p*-coum glc) in the obtained extracts. The same equation indicates that a positive linear effect on the quantity of malvidin-3-(6-*O*-*p*-coumaroyl) glucoside is obtained after using UAE parameters A (*p* < 0.0010), t (*p* < 0.0017) and Cw (*p* < 0.0001), including the quadratic effect of A (*p* < 0.0414) and interactions of t × Cw (*p* < 0.7400), A × t × Cw (*p* < 0.0455). A negative linear effect on the quantity of extracted malvidin-3-(6-*O*-*p*-coumaroyl) glucoside from the wine lees of the Merlot and Vranac varieties was provided by Ds (*p* < 0.0001) and the interactions A × t (*p* < 0.3454), A × Ds (*p* < 0.0156), A × Cw (*p* < 0.41743868), t × Ds (*p* < 0.0110), Ds × Cw (*p* < 0.0411). The analyzed extracts showed that the largest amount of malvidin-3-(6-*O*-*p*-coumaroyl) glucoside from the Merlot and Vranac varieties was obtained using high amplitudes and long extraction times when using a probe with a diameter of 22 mm. Results show that the wine lees extracts from the Vranac variety contain greater quantities of malvidin-3-(6-*O*-*p*-coumaroyl) glucoside.

Besides the detailed analysis of conditions for UAE of the compounds *trans*-resveratrol glucoside, *trans*-resveratrol, quercetin, kaempferol, petunidin-3-glucoside, malvidin-3-glucoside, malvidin-3-(6-*O*-acetyl) glucoside and malvidin-3-(6-*O*-*p*-coumaroyl) glucoside, which prevail in the wine lees and have high individual AOP, we further discovered the presence of other phenolic compounds that contribute to the overall AOP, but are primarily responsible for the astringent taste of red wines.

Malvidin-3-(6-*O*-p-coumaroyl) glucoside = 1.29 + 0.04 × A + 0.039 × t − 0.06 × Ds + 0.29 × Cw − 0.01 × A × t −0.03 × A × Ds − 0.01 × A × Cw − 0.03 × t × Ds + 0.00 × t × Cw − 0.01 × Ds × Cw + 0.02 × A^2^ + 0.03 × A × t × Cw b2 (*p* < 0.0001, R^2^ = 0.9750) (12)

### 3.3. Optimization of UAE Extraction Condition

Numerical optimization of the UAE for wine lees under research conditions, was performed by maximizing the response AOP_DPPH_ and AOP_FRAP_, in order to determine the optimal UAE conditions for obtaining extracts with the greatest AOP value, and responses were maximized for t-Res-3-*O*-glc (Merlot), *trans*-resveratrol (Vranac), quercetin, kaempferol, malvidin-3-glucoside, malvidin-3-(6-*O*-acetyl) glucoside, and malvidin-3-(6-*O*-*p*-coumaroyl) glucoside for wine lees from Merlot and Vranac varieties in order to obtain the most optimized UAE conditions for each wine lees separately.

**Figure 6:**
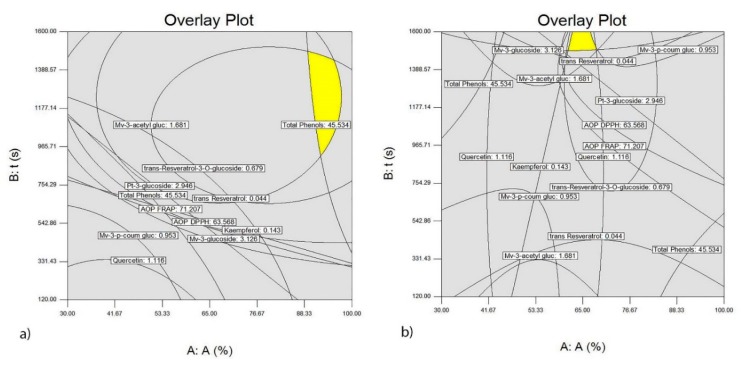
Optimal conditions of the ultrasound-assisted extraction of polyphenolic compounds from the wine lees of the Merlot variety which most significantly affect the AOP of the extracts using the ultrasonic probe with a diameter of 22 mm (**a**) and using the ultrasonic probe with a diameter of 40 mm (**b**).

**Figure 7:**
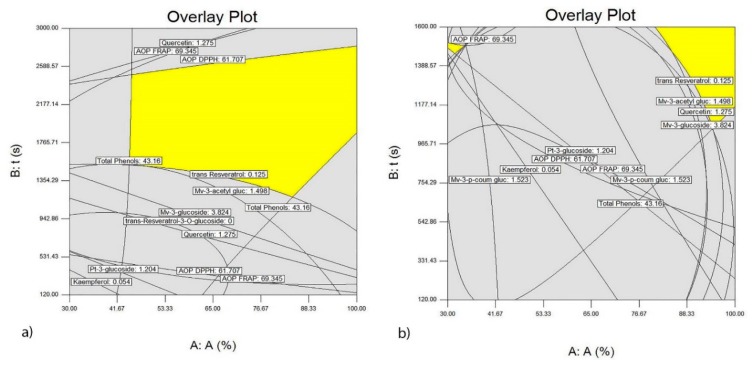
Optimal conditions of the ultrasound-assisted extraction of polyphenolic compounds from the wine lees of the Vranac variety which most significantly affect the AOP of the extracts using the ultrasonic probe with a diameter of 22 mm (**a**) and using the ultrasonic probe with a diameter of 40 mm (**b**).

The five best optimized extraction conditions are shown in Table 2 and Table 3, indicating that the extraction of the investigated variables from wine lees requires a probe with a diameter of 22 mm for both wine lees. Necessary extraction time for the lees of the Vranac variety is maximum, whereas for the wine lees from the Merlot variety the extraction time is shorter than 1361.33 s with desirability of 0.826. Based on the results obtained for each variety, a graphical optimization was performed, as shown in Figure 6a,b and Figure 7a,b, clearly showing the optimum area in which, the largest yields of the investigated polyphenolic compounds are achieved.

## 4. Discussion

Depending on the applied conditions of UAE, the AOP_DPPH_ in obtained extracts was enhanced by 76.39% and AOP_FRAP_ by 125.83% with respect to the CE obtained samples. AOP_DPPH_ and AOP_FRAP_ of wine lees extracts obtained using conventional extraction of the Vranac variety were higher than the Merlot variety, which may be a varietal characteristic or a consequence of the applied technologies. Equations (2) and (3), indicated that the highest AOP_DPPH_ and AOP_FRAP_ were found in the extracts obtained using the ultrasonic probe with a diameter of 22 mm, at a maximum amplitude and longest extraction time for both wine lees variety. In the wine lees extracts of the Vranac variety, a higher antioxidant capacity has been achieved than in the wine lees extracts of the Merlot variety.

The obtained results regarding AOP are in accordance with other authors [45,73], who also confirmed an increase of AOP in wine lees extracts obtained with non-thermal technologies compared to the CE. The obtained results regarding TPC are also in accordance with other authors [45,73]. They also confirmed an increase of TPC in wine lees extracts obtained with use of non-thermal technologies compared to the CE. Results show that *trans*-resveratrol glucoside was dominant in the extracts of wine lees from the Merlot while *trans*-resveratrol content is 20-fold lower. In comparison to the wine lees of the Merlot, *trans*-resveratrol glucoside (t-Res-3-*O*-glc) was not found in the wine lees of the Vranac, and the amount of *trans*-resveratrol was approximately three times greater compared to Merlot variety as shown in Table 2a,b and Table 3a,b.

In accordance with results from other authors [45] that successfully identified and extracted Quercetin from wine lees extracts, our results show that quercetin is present in similar quantities in lees from both varieties. The proportion of kaempferol is around three times greater in the wine lees of the Merlot variety as compared to Vranac variety. Moreover, in the samples that were analyzed, anthocyanin monomers responsible for the wine’s color were obtained from the investigated grape varieties.

Likewise, Pérez-Serradilla and Luque de Castro [45] successfully identified and extracted malvidin-3-(6-*O*-acetyl) glucoside from wine lees extracts, and found that malvidin-3-glucoside (Mv-3-glucoside) and petunidin-3-glucoside (Pt-3-glc) were the predominant anthocyanins in Merlot samples. There are twice as much malvidin-3-(6-*O*-acetyl) glucoside, but malvidin-3-(6-*O*-*p*-coumaroyl) glucoside was three times lower than Mv-3-glucoside. Mv-3-glucoside prevails in the extract from the Vranac as compared to Merlot. The proportion of malvidin-3-(6-*O*-acetyl) glucoside, malvidin-3-(6-*O*-*p*-coumaroyl) glucoside and Pt-3-glc in samples is two and half times less then Mv-3-glucoside but in similar quantities. Based on the results, it is concluded that for the greatest quantity of investigated polyphenolic compounds from wine less of the Merlot variety, the best most optimized parameters are use of probe with a diameter of 22 mm under the following UAE conditions: Applied amplitude of the ultrasonic processor ranging from 89.28% to 100% and an extraction time of between 921.81 s to 1492.15 s. To obtain the greatest quantity of investigated polyphenolic compounds from wine lees the Vranac variety, best optimized parameters are probe with a diameter of 22 mm under the following UAE conditions, applied amplitude of the ultrasonic processor ranging from 44.86% to 93.64%, and an extraction time of between 1176.81 s to 1500 s. The confirmed results are in accordance with previously reported findings which also depicted a higher yield of TPC, AOP and polyphenols such as quercetin, malvidin-3-glucoside (Mv-3-gluc), myricetin in the range from 19−20% when compared to the CE [45,73].

## 5. Conclusions

It is evident from the findings obtained in this work that ultrasound-assisted extraction (UAE) of polyphenols from lees of the Merlot variety (five best optimized extraction conditions) results in significantly higher yields of bioactive compounds in the extracts compared to conventional extraction (CE). As per the DPPH assay, it is observed that UAE of the Merlot variety enhanced AOP from 76.05% to 76.39% when compared to the CE. Similarly, AOP FRAP, total phenols, *trans*-resveratrol glucoside, *trans*-resveratrol, quercetin, kaempferol, petunidin-3-glucoside, malvidin-3-glucoside, malvidin-3-(6-*O*-acetyl) glucoside and malvidin-3-(6-*O*-*p*-coumaroyl) glucoside extraction was enhanced significantly in wine lees from both the varieties after using UAE. Therefore, it is concluded that ultrasound processing can be successfully used to enhance the extraction of bioactive compounds from wine lees, a by-product of the wine industry. Considering that very limited studies have been reported about the composition and bioactivity of wine lees extracts, as well as the extraction of phenolics from lees, a more detailed investigation could pave newer ways to utilize in wine industry waste effectively.

## Figures and Tables

**Table:** (**a**)

A	t	Ds	AOP DPPH	AOP FRAP	TPC	*trans*-resv-3-*O*-gluc	*trans* Resveratrol	Quercetin	Kaempferol	Pt-3-Glucoside	Mv-3-Glucoside	Mv-3-acetyl gluc	Mv-3-*p*-coum gluc
38.79	322.10	22	56.43 ± 0.31	58.05 ± 1.21	56.52 ± 1.11	0.61 ± 0.00	0.04 ± 0.00	1.09 ± 0.02	0.13 ± 0.01	2.44 ± 0.05	2.46 ± 0.04	1.36 ± 0.01	0.79 ± 0.01
81.21	322.10	22	64.17 ± 0.22	68.76 ± 1.13	37.64 ± 1.52	0.68 ± 0.00	0.04 ± 0.00	1.23 ± 0.01	0.15 ± 0.02	3.00 ± 0.09	2.91 ± 0.01	1.64 ± 0.02	1.11 ± 0.02
38.79	1297.90	22	68.09 ± 0.11	76.99 ± 0.98	32.45 ± 1.21	0.69 ± 0.01	0.04 ± 0.01	1.25 ± 0.03	0.15 ± 0.01	3.09 ± 0.08	3.33 ± 0.02	1.57 ± 0.01	1.08 ± 0.03
81.21	1297.90	22	78.30 ± 0.61	92.74 ± 0.96	39.01 ± 1.09	0.78 ± 0.01	0.05 ± 0.01	1.37 ± 0.01	0.16 ± 0.03	3.41 ± 0.01	3.68 ± 0.03	1.74 ± 0.03	1.17 ± 0.01
30.00	810.00	22	60.98 ± 0.21	66.97 ± 0.99	54.30 ± 0.89	0.64 ± 0.00	0.04 ± 0.00	1.21 ± 0.02	0.15 ± 0.03	2.96 ± 0.11	3.22 ± 0.01	1.64 ± 0.01	1.00 ± 0.02
90.00	810.00	22	72.94 ± 0.11	82.29 ± 1.12	49.57 ± 0.75	0.72 ± 0.01	0.04 ± 0.01	1.31 ± 0.03	0.16 ± 0.01	3.22 ± 0.09	3.47 ± 0.04	1.64 ± 0.01	1.14 ± 0.01
60.00	120.00	22	48.39 ± 0.40	52.45 ± 1.50	50.42 ± 0.49	0.63 ± 0.00	0.04 ± 0.01	1.12 ± 0.01	0.13 ± 0.01	2.63 ± 0.08	2.99 ± 0.05	1.45 ± 0.01	1.03 ± 0.02
60.00	1500.00	22	76.98 ± 0.55	89.90 ± 1.11	43.74 ± 0.29	0.75 ± 0.00	0.05 ± 0.00	1.32 ± 0.02	0.17 ± 0.02	3.09 ± 0.11	3.41 ± 0.04	1.67 ± 0.20	1.12 ± 0.01
60.00	810.00	22	67.61 ± 0.51	77.31 ± 2.14	42.40 ± 1.93	0.67 ± 0.01	0.04 ± 0.00	1.21 ± 0.01	0.14 ± 0.01	3.01 ± 0.08	3.29 ± 0.04	1.68 ± 0.02	1.06 ± 0.01
38.79	322.10	40	48.64 ± 0.02	55.39 ± 2.10	49.13 ± 1.01	0.63 ± 0.01	0.04 ± 0.01	1.16 ± 0.01	0.15 ± 0.00	2.85 ± 0.03	2.97 ± 0.11	1.59 ± 0.11	1.02 ± 0.02
81.21	322.10	40	53.43 ± 0.05	58.32 ± 2.11	42.36 ± 0.99	0.68 ± 0.01	0.04 ± 0.02	1.10 ± 0.01	0.16 ± 0.01	3.07 ± 0.02	3.13 ± 0.09	1.69 ± 0.11	1.02 ± 0.02
38.79	1297.90	40	59.97 ± 0.20	66.48 ± 1.51	48.68 ± 0.84	0.64 ± 0.00	0.04 ± 0.01	1.09 ± 0.03	0.14 ± 0.01	2.87 ± 0.01	3.23 ± 0.09	1.61 ± 0.09	1.03 ± 0.01
81.21	1297.90	40	65.31 ± 0.31	82.36 ± 1.31	46.18 ± 0.85	0.68 ± 0.02	0.04 ± 0.00	1.03 ± 0.02	0.14 ± 0.02	2.41 ± 0.04	2.45 ± 0.05	1.46 ± 0.80	0.93 ± 0.01
30.00	810.00	40	55.93 ± 0.33	70.57 ± 1.89	46.49 ± 0.77	0.60 ± 0.01	0.03 ± 0.00	1.00 ± 0.02	0.13 ± 0.01	2.38 ± 0.02	2.64 ± 0.09	1.18 ± 0.11	0.94 ± 0.02
90.00	810.00	40	62.16 ± 0.11	71.25 ± 2.05	57.24 ± 0.22	0.66 ± 0.00	0.04 ± 0.01	0.85 ± 0.01	0.13 ± 0.03	2.90 ± 0.02	3.11 ± 0.11	0.32 ± 0.05	1.04 ± 0.01
60.00	120.00	40	38.93 ± 0.22	41.97 ± 1.13	51.92 ± 0.56	0.64 ± 0.01	0.05 ± 0.01	1.06 ± 0.03	0.14 ± 0.04	2.46 ± 0.01	2.74 ± 0.10	1.52 ± 0.09	0.94 ± 0.02
60.00	1500.00	40	63.99 ± 0.31	69.25 ± 2.98	48.31 ± 0.78	0.68 ± 0.01	0.04 ± 0.00	1.15 ± 0.02	0.15 ± 0.00	2.98 ± 0.02	3.15 ± 0.11	1.66 ± 0.08	0.99 ± 0.01
60.00	810.00	40	58.34 ± 0.62	66.39 ± 3.15	52.63 ± 1.08	0.67 ± 0.02	0.04 ± 0.00	1.16 ± 0.02	0.14 ± 0.00	2.92 ± 0.03	2.94 ± 0.16	1.52 ± 0.14	0.95 ± 0.09
CE	3600.00		44.21 ± 0.33	42.52 ± 1.13	32.95 ± 1.09	0.60 ± 0.01	0.030 ± 0.00	1.01 ± 0.02	0.10 ± 0.02	2.26 ± 0.03	2.39 ± 0.09	1.36 ± 0.09	0.93 ± 0.01

**Table:** (**b**)

A	t	Ds	AOP _DPPH_	AOP _FRAP_	TPC	*trans*-resv-3-*O*-gluc	*trans* Resveratrol	Quercetin	Kaempferol	Pt-3-Glucoside	Mv-3-Glucoside	Mv-3-acetyl gluc	Mv-3-*p*-coum gluc
38.79	322.10	22	59.86 ± 0.11	68.83 ± 1.33	44.92 ± 0.78	0.00 ± 0.00	0.11 ± 0.01	1.17 ± 0.02	0.06 ± 0.01	1.27 ± 0.03	3.40 ± 0.05	1.30 ± 0.02	1.62 ± 0.02
81.21	322.10	22	67.20 ± 0.25	76.28 ± 1.25	41.34 ± 0.63	0.00 ± 0.00	0.12 ± 0.02	1.22 ± 0.03	0.06 ± 0.01	1.37 ± 0.02	3.70 ± 0.06	1.45 ± 0.01	1.64 ± 0.01
38.79	1297.90	22	70.07 ± 0.10	79.82 ± 1.29	40.48 ± 0.22	0.00 ± 0.00	0.11 ± 0.00	1.29 ± 0.02	0.05 ± 0.02	1.41 ± 0.03	3.78 ± 0.01	1.39 ± 0.01	1.66 ± 0.02
81.21	1297.90	22	79.76 ± 0.05	96.45 ± 1.34	43.70 ± 0.45	0.01 ± 0.00	0.13 ± 0.02	1.42 ± 0.04	0.08 ± 0.03	1.52 ± 0.01	4.24 ± 0.02	1.63 ± 0.02	1.80 ± 0.01
30.00	810.00	22	65.39 ± 0.09	73.34 ± 1.02	32.34 ± 0.36	0.00 ± 0.00	0.11 ± 0.01	1.22 ± 0.03	0.07 ± 0.01	1.30 ± 0.02	3.31 ± 0.03	1.36 ± 0.03	1.61 ± 0.02
90.00	810.00	22	73.58 ± 0.24	90.62 ± 1.27	32.61 ± 0.56	0.00 ± 0.00	0.11 ± 0.00	1.34 ± 0.02	0.06 ± 0.00	1.43 ± 0.04	3.96 ± 0.04	1.51 ± 0.02	1.74 ± 0.01
60.00	120.00	22	51.00 ± 0.11	56.14 ± 1.08	34.20 ± 0.68	0.00 ± 0.00	0.09 ± 0.01	1.11 ± 0.03	0.06 ± 0.01	1.15 ± 0.01	3.32 ± 0.05	1.18 ± 0.01	1.55 ± 0.02
60.00	1500.00	22	76.47 ± 0.22	96.75 ± 1.04	49.16 ± 0.71	0.00 ± 0.00	0.13 ± 0.02	1.35 ± 0.02	0.07 ± 0.01	1.41 ± 0.02	4.02 ± 0.01	1.54 ± 0.00	1.74 ± 0.00
60.00	810.00	22	71.68 ± 0.32	85.93 ± 1.44	48.09 ± 0.84	0.00 ± 0.00	0.11 ± 0.01	1.28 ± 0.03	0.06 ± 0.00	1.35 ± 0.02	3.75 ± 0.07	1.37 ± 0.02	1.66 ± 0.02
38.79	322.10	40	52.73 ± 0.22	55.63 ± 2.10	43.35 ± 1.21	0.00 ± 0.00	0.10 ± 0.00	1.14 ± 0.03	0.05 ± 0.01	1.16 ± 0.01	3.11 ± 0.02	1.24 ± 0.05	1.51 ± 0.02
81.21	322.10	40	58.77 ± 0.66	66.56 ± 1.57	37.09 ± 1.51	0.00 ± 0.00	0.11 ± 0.01	1.18 ± 0.01	0.07 ± 0.02	1.22 ± 0.02	3.26 ± 0.02	1.31 ± 0.04	1.55 ± 0.01
38.79	1297.90	40	62.74 ± 0.22	70.80 ± 1.45	43.53 ± 1.32	0.00 ± 0.00	0.11 ± 0.01	1.18 ± 0.02	0.06 ± 0.01	1.29 ± 0.01	3.37 ± 0.05	1.39 ± 0.04	1.57 ± 0.02
81.21	1297.90	40	69.40 ± 0.45	81.12 ± 2.01	54.06 ± 1.52	0.00 ± 0.00	0.12 ± 0.00	1.24 ± 0.04	0.07 ± 0.01	1.41 ± 0.02	3.71 ± 0.01	1.43 ± 0.03	1.61 ± 0.02
30.00	810.00	40	57.98 ± 0.25	64.99 ± 1.55	47.34 ± 1.03	0.00 ± 0.00	0.11 ± 0.01	1.18 ± 0.03	0.05 ± 0.02	1.27 ± 0.03	3.34 ± 0.02	1.37 ± 0.04	1.57 ± 0.01
90.00	810.00	40	64.14 ± 0.29	71.33 ± 1.36	37.84 ± 1.05	0.00 ± 0.00	0.11 ± 0.01	1.19 ± 0.02	0.06 ± 0.01	1.35 ± 0.01	3.63 ± 0.03	1.38 ± 0.03	1.59 ± 0.01
60.00	120.00	40	40.74 ± 0.35	44.24 ± 1.32	39.66 ± 1.10	0.00 ± 0.00	0.10 ± 0.00	1.08 ± 0.01	0.05 ± 0.00	1.10 ± 0.01	2.98 ± 0.00	1.21 ± 0.05	1.56 ± 0.01
60.00	1500.00	40	65.89 ± 0.38	73.05 ± 1.87	57.43 ± 1.21	0.00 ± 0.00	0.11 ± 0.00	1.24 ± 0.04	0.06 ± 0.00	1.34 ± 0.02	3.57 ± 0.04	1.19 ± 0.01	1.56 ± 0.02
60.00	810.00	40	59.95 ± 0.88	67.26 ± 2.18	48.86 ± 1.71	0.00 ± 0.00	0.09 ± 0.00	1.09 ± 0.03	0.05 ± 0.00	1.04 ± 0.00	2.71 ± 0.05	1.06 ± 0.08	1.44 ± 0.02
CE	3600.00		49.72 ± 0.22	50.39 ± 1.08	41.22 ± 1.03	0.00 ± 0.00	0.09 ± 0.01	1.00 ± 0.01	0.04 ± 0.00	0.97 ± 0.03	2.36 ± 0.02	1.1 ± 0.03	1.40 ± 0.01

A, amplitude; t, time; Ds, probe diameter; AOP (DPPH) and AOP (FRAP), antioxidant potential; TPC, total phenolic content; trans-resv-3-*O*-gluc: trans-resveratrol-3-*O*-glucoside; Pt-3-glucoside, petunidin-3-glucoside; Mv-3-glucoside, malvidin-3-glucoside; Mv-3-acetyl gluc, malvidin-3-(6-*O*-acetyl) glucoside; Mv-3-p-coum gluc, malvidin-3-(6-*O*-p-coumaroyl) glucoside; Mv, Malvidin; Pt, Petunidin; CE, results of Classical extraction.

**Table:** (**a**)

A	t	Ds	AOP DPPH	AOP FRAP	TPC	*trans*-resv- 3-*O*-gluc	*trans* Resveratrol	Quercetin	Kaempferol
90.000	1361.36	22	76.05	122.98	38.67	31.56	36.36	41.43	68.69
90.000	1364.32	22	76.07	123.06	38.69	31.73	36.36	41.53	68.69
90.000	1468.60	22	76.39	125.33	39.28	34.05	36.36	43.33	71.72
89.999	1313.80	22	75.71	121.66	38.37	30.57	36.36	40.64	67.68
90.000	1497.93	22	76.38	125.83	39.45	34.72	36.36	43.83	72.73

**Table:** (**b**)

A	t	Ds	Pt-3-Glucoside	Mv-3-Glucoside	Mv-3-acetyl gluc	Mv-3-*p*-coum gluc	Desirability
90.00	1361.34	22	41.92	49.10	25.98	26.88	0.826
90.00	1364.32	22	41.97	49.10	25.90	26.88	0.826
90.00	1468.59	22	42.90	48.01	24.35	26.77	0.824
89.99	1313.79	22	41.52	49.43	26.49	26.88	0.824
90.00	1497.93	22	43.12	47.63	23.84	26.77	0.823

A, amplitude; t, time; Ds, probe diameter; AOP _(DPPH)_ and AOP _(FRAP),_ antioxidant potential; TPC, total phenolic content; trans-resv-3-*O*-gluc: *trans*-resveratrol-3-*O*-glucoside; A, amplitude; t, time; Ds, probe diameter; Pt-3-glucoside, petunidin3-glucoside; Mv-3-glucoside, malvidin-3-glucoside; Mv-3-acetyl gluc, malvidin-3-(6-*O*-acetyl) glucoside; Mv-3-*p*-coum gluc, malvidin-3-(6-*O*-*p*-coumaroyl) glucoside.

**Table:** (**a**)

A	t	Ds	AOP DPPH	AOP FRAP	TPC	*trans*-resv- 3-*O*-gluc	*trans* Resveratrol	Quercetin	Kaempferol
90.00	1499.98	22	62.19	104.79	4.620	300.00	45.75	42.23	67.44
90.00	1467.48	22	62.20	104.33	3.54	300.00	44.68	42.03	67.44
86.17	1499.99	22	61.01	103.10	9.61	300.00	43.62	41.24	65.12
85.57	1499.98	22	60.83	102.81	10.31	300.00	43.62	41.04	65.12
81.49	1500.00	22	59.57	100.78	14.41	300.00	41.49	39.94	65.12

**Table:** (**b**)

A	t	Ds	Pt-3-Glucoside	Mv-3-Glucoside	Mv-3-acetyl gluc	Mv-3-*p*-coum gluc	Desirability
90.00	1499.99	22	64.95	89.17	49.74	34.93	0.828
90.00	1467.48	22	64.33	88.03	49.04	34.50	0.826
86.17	1499.99	22	63.61	87.94	46.77	33.21	0.822
85.50	1499.98	22	63.40	87.73	46.34	33.00	0.821
81.49	1500.00	22	61.86	86.38	43.46	31.29	0.812

A, amplitude; t, time; Ds, probe diameter; AOP (DPPH) and AOP (FRAP), antioxidant potential; TPC, total phenolic content; trans-resv-3-*O*-gluc: *trans*-resveratrol-3-*O*-glucoside; A, amplitude; t; time; Ds, probe diameter; Pt-3-glucoside, petunidin3-glucoside; Mv-3-glucoside, malvidin -3-glucoside; Mv-3-acetyl gluc, malvidin-3-(6-*O*-acetyl) glucoside; Mv-3-*p*-coum gluc, malvidin-3-(6-*O*-*p*-coumaroyl) glucoside.
